# Characterization of Breakfast Cereals Available in the Mexican Market: Sodium and Sugar Content

**DOI:** 10.3390/nu9080884

**Published:** 2017-08-16

**Authors:** Claudia Nieto, Sofia Rincon-Gallardo Patiño, Lizbeth Tolentino-Mayo, Angela Carriedo, Simón Barquera

**Affiliations:** 1 Instituto Nacional de Salud Pública, Av. Universidad 655, Col. Santa María Ahuacatitlán, Cuernavaca C.P 62100, Morelos, Mexico; claudia.nieto@insp.mx (C.N.); sbarquera@insp.mx (S.B.); 2 Virginia Tech, 223 Wallace Hall, Blacksburg, VA 24061, USA; sofiargp@vt.edu; 3 London School of Hygiene & Tropical Medicine, Keppel St, Bloomsbury, London WC1E 7HT, UK; angela.carriedo@lshtm.ac.uk

**Keywords:** breakfast cereals, edible grain, nutrition labelling, claims

## Abstract

Preschool Mexican children consume 7% of their total energy intake from processed breakfast cereals. This study characterized the nutritional quality and labelling (claims and Guideline Daily Amount (GDA)) of the packaged breakfast cereals available in the Mexican market. Photographs of all breakfast cereals available in the 9 main food retail chains in the country were taken. The nutrition quality of cereals was assessed using the United Kingdom Nutrient Profiling Model (UKNPM). Claims were classified using the International Network for Food and Obesity/non-communicable Diseases Research, Monitoring and Action Support (INFORMAS) taxonomy and the GDA was defined according to the Mexican regulation, NOM-051. Overall, a total of 371 different breakfast cereals were analysed. The nutritional profile showed that 68.7% were classified as “less healthy”. GDAs and claims were displayed more frequently on the “less healthy” cereals. Breakfast cereals within the “less healthy” category had significantly higher content of energy, sugar and sodium (*p* < 0.001). Most of the claims were displayed in the “less healthy” cereals (*n* = 313). This study has shown that there is a lack of consistency between the labelling on the front of the pack and the nutritional quality of breakfast cereals.

## 1. Introduction

The consumption of ultra-processed foods has become a common practice in the whole world [1,2,3], with this consumption pattern also occurring in Latin America and Mexico [3,4]. In Mexico, the second highest contribution to total energy intake has come from products that are high in saturated fat, added sugar and sodium [4]. A total of 58% of the calories consumed by Mexicans came from packaged foods and beverages [5]. Breakfast cereals, ready-to-eat cereals or sweetened cereals are considered ultra-processed food products with high amounts of energy, saturated fat, sugar and sodium [6,7,8]. Evidence suggests that an excessive intake of calories and added sugars from packaged food and sweetened beverages has contributed to the rapid growth of obesity worldwide [9]. The proportion of obese adults and children has increased in both developed and developing countries [10]. The latest Mexican National Health and Nutrition Survey states that the prevalence of being overweight and obese reached 72.5% in adults, while the combined prevalence is 33.2% for children [11]. 

In a similar way, consuming too much sodium contributes to a range of adverse health outcomes [12,13,14]. Excessive sodium intake is a dietary risk factor that contributes to hypertension, cardiovascular diseases and death [12,13,14,15]. In 2013, the World Health Organization Global Action Plan of for the Prevention and Control of Non-Communicable Diseases [16] set a target to reduce the mean population intake of sodium by 30%. Some studies [17,18,19,20] have reported that most processed foods have a high sodium content. Another study has suggested that one of the key actions to reduce the total population’s salt intake is a 54% reduction in the sodium content of ready-to-eat breakfast cereals, along with other reductions, such reducing the sodium content in other processed foods, reducing sodium in foods consumed away from home, and reducing discretionary salt use. All of these changes would result in the achievement of the WHO sodium target [21].

One of the main dietary patterns for breakfast among Mexican children consists of cereal with milk; 6% of Mexican children exclusively consume ready-to-eat cereals with milk for breakfast [22]. The Mexican National Health and Nutrition Survey of 2012 indicates that between 42% and 49% of children aged <2 years consumed sweetened cereals [23]. Another study mentioned that the highest dietary energy contribution (33%) came from minimally processed cereals [24]. An observational study reported that 7% of the total energy intake of preschool Mexican children was from processed breakfast cereals [25]. Therefore, breakfast cereals substantially contribute to the daily energy and nutrient intakes of the Mexican population. An international survey estimated that Mexican breakfast cereals provided at least 600 mg sodium/100 g [26], which is 1.5 times the UK maximum target for breakfast cereals (the average target is 235 mg) [27]. Furthermore, whilst there is no recent nationally representative study, a 1998 study in a small sample of the normotensive population in the northern region of Mexico estimated the consumption of salt was 9.4 g [28]. A more recent study [29] in 2017 showed that 20% of the population adds salt to their meals. The same population was reported to add salt five days a week, twice a day. A contribution of 44% of the sodium consumed in the country came from breads, meats, pizzas, soups, sandwiches, cheese and snacks. A study conducted in healthy participants that evaluated 24-h urinary excretion reported that the sodium intake was higher than the WHO recommendations [30]. 

A systematic review concluded that consuming ready-to-eat cereals is associated with a higher sugar intake [31]. In an experimental study, high-sugar cereals were found to increase the total sugar consumption of children and decrease the nutrition quality of their breakfast [8]. Breakfast cereals have typically been marketed as healthy products. Nevertheless, it has been documented that such products feature nutritional or health claims, promotional characters and/or premium offers as a marketing strategy, which are frequently oriented towards children [32,33]. In Australia, breakfast cereals are among the food categories with the highest percentage of products (54%) carrying health or nutritional claims [34]. According to Dixon et al. [35], nutrient content claims and sports celebrity endorsements influence preferences towards energy-dense and nutrient-poor food products displaying these claims and endorsements.

Several strategies to tackle obesity have been recognized worldwide that aim to improve diets. The front of package labelling (FOPL) system for food products is a tool that informs consumers about the nutritional content of foods in an easy and simple way [36,37]. These systems are being regulated by governments as a public health strategy to influence the population into adopting healthier diets, such as Bolivia, Chile, Ecuador, United Kingdom, Mexico, New Zealand and Australia [38,39].

### Context of Front of Package Labelling Regulation in Mexico

Before the FOPL became mandatory in 2014, the largest consortium of food manufacturers in Mexico decided to place a voluntary FOPL system called the GDA (Guideline Daily Amount) on packaged foods as a means of self-regulation. However, evidence has found that this type of FOPL system is not well understood by Mexican consumers [40,41]. Such labelling had reference values higher than the WHO recommendations on certain nutrients, including sugar and sodium. The current mandatory food labelling launched in 2014 is regulated by the Official Mexican Norm 051 [42], which provides manufacturers with the needed information to place the front of package labels on their food products. Other regulations have been implemented in Mexico since 1988. The Official Mexican Norm 086 [43] (NOM-086 by their initials in Spanish) regulates the presence of claims in the front of the package. Therefore, all nutritional declarations should be submitted to the NOM-086. 

Given the significant contribution of breakfast cereals to the Mexican diet as well as the lack of strong policies for claims and food promotion, it is important to assess the quality of breakfast cereals and to identify current practices of package labelling. The aim of this study was to characterize the nutritional quality of breakfast cereals using the Nutrient Profiling Model of the United Kingdom (UKNPM) [44] and to characterize the labelling (use of claims and GDA) in such products.

## 2. Materials and Methods

### 2.1. Sampling 

This is a cross-sectional study conducted in four different cities of Mexico from November 2013 until December 2014. Data were collected with photos of breakfast cereals (*n* = 434) taken from the eight main food retail chains in the country. A two-staged sampling design was used to select the stores from which to collect breakfast cereal data from in order to have a variety of food-retailer stores from different socio-economic areas in four cities in Mexico. The stores were mapped using a geo-reference system to determine the AGEB (Area Geoestadísticas Básicas-basic geo-statistical area) of these locations. AGEBs are delineated urban areas with 25,000 inhabitants or more, which are used to locate specific socio-demographic circumstances, such as living conditions, use of the land and so on. They are proxy estimations of the socio-demographic characteristics of areas in each city. The supermarkets in each AGEB were randomly selected to be proportional to the distribution of the three levels of marginalization defined by the National Institutes of Statistics and Geography on a scale of low, middle and high. To cover a broad sample of breakfast cereals, we included different retailers from urban areas of each city. Fieldworkers visited a total of 9 stores and in each store, photos were captured of every different cereal product on the shelf at the time of the visit. The sampling strategy for cereals was for convenience, but it allowed an extensive coverage of the stocked cereals in Mexico.

### 2.2. Ethical Approval 

This study was evaluated and approved by the Research, Ethics and Biosafety Committees of the National Institute of Public Health of Mexico (ethical approval number: 1153). Before conducting the study, the research team asked for permission from the supermarket’s manager to access the stores and take photos of available breakfast cereals in the country.

### 2.3. Data Collection

Data from all available breakfast cereals in the selected supermarkets were collected. Trained nutritionists took photographs of available cereals in supermarkets using a smartphone. Six photographs of each breakfast cereal were taken (front of package, back of package, GDA, price, nutrient content and promotion; if applicable). Duplicated products or products with missing data and illegible photographs were not analysed (*n* = 63). Therefore, we analysed the information of (*n* = 371) breakfast cereals. To create the variables, data were directly obtained from the photographs of the cereal package and transcribed to an excel spreadsheet. The personnel who captured the data followed a standardized operation procedure created by the researchers of the Mexican National Institute of Public Health. We included information, such as product name, brand, price, GDA, claims, serving size, nutrition content and location of the supermarket. The GDA was defined according to the Mexican regulation, NOM-051 [42], which states that a food product must display nutritional information on the front of the package. The nutrients of concern are those nutrients that may pose a substantial public health concern due to overconsumption. These include energy, saturated fat, sugar and sodium [45].

### 2.4. Nutrition Quality of Breakfast Cereals 

Nutrient content was analysed per 100 g of each product. The analysed nutrients were energy (kcal), saturated fat (g), sugar (g), sodium (mg), fibre (g) and protein (g). The nutrition quality of cereals was assessed using the United Kingdom Nutrient Profiling Model (UKNPM) [44]. The model uses a simple scoring system where points are allocated based on the nutrient content of 100 g of a food or drink. Points are awarded for ‘A’ nutrients (energy, saturated fat, total sugar and sodium) and for ‘C’ nutrients (fruit, vegetables and nut content, fibre and protein). The score for ‘C’ nutrients is then subtracted from the score for ‘A’ nutrients to give the final nutrient profile score [44]. This model provides an assessment of the overall nutrition composition. Products with a score greater or equal than four were considered “less healthy”, while a score less than four were considered “healthy” products. This categorization was used for the sample of breakfast cereals to categorize the quality of such products available for consumers in different areas of the country.

### 2.5. Claims 

Claims are defined as any representation which states, suggests or implies that a food has particular characteristics relating to its origin, nutritional properties, nature, production, processing, composition and any other quality [46]. In order to categorize the claims, the exact text displayed in the front of the package of cereals was typed into the database. Claims were coded and classified using the International Network for Food and Obesity/non-communicable Diseases Research, Monitoring and Action Support (INFORMAS) taxonomy [47], which is shown in Table 1. Claims were divided into three main categories and their subcategories: (1) nutritional claims (health-related claim and nutrient claim); (2) health claims (general health claim, nutrient and other function claim as well as reduction of disease risk claim); and (3) other claims (environment-related claim and other health-related claims). A single food product could display several types of claims. The format of the claim was considered, including whether it was written text, numerical or symbolic. Products with a combination of numerical and written text formats within the same claim were coded as a numerical format. 

### 2.6. Statistical Analysis

Proportions, medians and standard deviations (SD) were used to summarize descriptive data for the nutritional content of the products. Nutrient values were standardized by the amount per 100 g of product. The prevalence ratio was calculated to find out how more likely is to find FOP labels and claims in less healthy products. Products were categorized into two categories: “healthy” and “less healthy”. This categorization was based in the UKNPM. Mann–Whitney U Tests was used to compare nutritional content of the two categories as well as to make comparisons between the labelling of the packages (products carrying a claim and products without claims; in addition to products carrying a GDA and products without GDA). A Chi^2^ test was used to assess differences between the UKNPM scores for individual nutrients. A p less than 0.05 was considered statistically significant. Data were analysed using the Statistical Package STATA (Version 12.0).

## 3. Results

A total of 371 breakfast cereals were analysed, which represented a total of 84 brands. The nutritional profile showed that 31.3% of the cereals were under the “healthy” classification, while 68.7% were classified as “less healthy”. The average energy content in the sample of breakfast cereals was 374.4 ± 122.2 kcal. These cereals had on average 1 ± 1.7 g of saturated fat, 26.6 ± 14.1 g of sugar and 450 ± 225.5 mg of sodium.

GDAs in FOPL were displayed more frequently in “less healthy” cereals compared to “healthy cereals”. From the sample, 23% of the GDAs were displayed on “healthy” cereals, while 77% were displayed on “less healthy” cereals. Similarly, the claims were displayed more frequently in “less healthy” cereals (68%). Table 2 displays the mean UKNPM score of the nutrients of interest (energy, saturated fat, sugar and sodium). Significant differences between “healthy” and “less healthy” cereals were found for energy (*p* = 0.02), sugar (*p* < 0.001) and sodium (*p* < 0.001).

**Figure 1:**
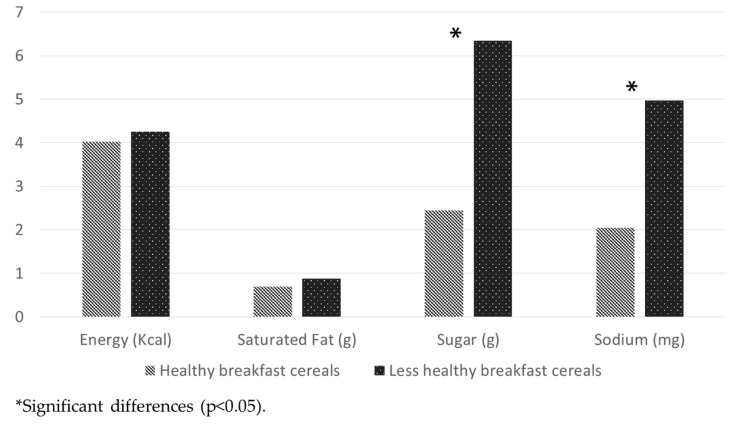
United Kingdom Nutrient Profiling Model scores of breakfast cereals in the Mexican market.

Breakfast cereals under the “less healthy” category had a significantly higher content of energy, sugar and sodium (*p* < 0.001). In addition, the median number of calories in the “healthy” category was lower compared to the median number in “less healthy” cereals (*p* < 0.001). When comparing the nutritional content of cereals with claims and no-claims, there were significant differences in energy content (*p* = 0.02). For other nutrients, such as saturated fat, sugar and sodium, the nutritional content was almost the same with no significant differences. In Figure 1, “less healthy” cereals have mean scores of sugar (6.3) and sodium (4.9) that were almost double those for the “healthy” cereals.

### 3.1. Labelling of the Breakfast Cereals (GDAs and Claims in the Front of Package Labelling)

Breakfast cereals with GDA in the FOPL are 1.4 times more likely to be classified as ‘less healthy’ in comparison to cereals with no GDA in the label (95% CI = 1.19–1.65). Cereals that displayed the GDA in the front of the package had a higher content of sugar (*p* < 0.004) and sodium (*p* < 0.001) as well as a higher UKNPM score (*p* = 0.001). However, cereals that displayed GDA had less calories (*p* = 0.001) compared to cereals that did not displayed any GDA (Table 2). Not all of the sample of breakfast cereals displayed the GDA system, with only (*n* = 228) breakfast cereals displaying the GDA on the front of the package. The “less healthy” category of cereals displayed the GDA more frequently (*n* = 176, 77.2%), while healthy cereals displayed the GDA less frequently (*n* = 52, 23%) (Table 3). 

### 3.2. Displayed Claims on Breakfast Cereals 

A total of 282 (76%) breakfast cereals displayed 587 claims, with an average of 2 claims per package. Furthermore, a single product could display up to 9 different claims on the front of the package. From the overall claims displayed in breakfast cereals, nutritional claims represented 86%, while health claims comprised 4% and other claims were on 10% (Table 3). We found that most of the nutritional content claims were commonly about antioxidants/vitamins/minerals (55%), followed by fibre (19%) and fats (10%). We found only two claims about sodium (1%). Claims about cholesterol (3.3%) and energy (1.4%) were mostly displayed on the “less healthy” cereals. The breakfast cereals categorized as “healthy” displayed health-related ingredient claims (22.7%), nutrient content claims (70.7%) and nutrient comparative claims (6.7%). However, none of the cereals under the healthy category displayed claims about having more calcium or more fibre in the package. We found a higher proportion of health-related ingredient claims and nutritional content claims on the “less healthy” category of cereals. For the “less healthy” cereals, 70.5% featured nutrient content claims, 25.9% featured health-related ingredient claims and 3.6% featured nutrient comparative claims. For the nutrient comparative claims, over 68% were made about antioxidants/vitamins/minerals (e.g., fortified with vitamins and minerals; as well as great source of iron). Most of the claims were presented in the written text format (71%), followed by numerical (26%) and symbolic (2%) types of claims. All the symbolic format of claims (*n* = 14) were displayed on the healthy category of cereals.

## 4. Discussion

This cross-sectional study is the first study that explores the nutritional quality, labelling and different types of claims displayed on breakfast cereals in the Mexican market. Approximately 69% of the cereals in the Mexican market fall into the category of being “less healthy”. However, this information is not easy to interpret on the food label and is frequently not available to consumers. Many “less healthy” cereals carried nutritional or health messages. Nevertheless, the nutritional content of breakfast cereals was significantly higher for the nutrients of concern, such as energy, sugar and sodium, compared to those cereals categorised as “healthy”.

As national data revealed one of the most important breakfast food patterns includes the intake of ready to eat cereal with milk [22], overconsumption of cereals filled with nutrients of interest might represent a risk for obesity, diabetes, hypertension and cardiovascular diseases. The Nutrient Intake Recommendations for the Mexican population estimates that the breakfast intake for preschool children should be around 325 calories [48]. However, a single serving of 100 g of breakfast cereal contributes an average of 362.8 calories, which is higher than the breakfast recommendation. 

Some studies have found that breakfast cereals contributed to nutritional requirements, which is explained by food fortification [49,50]. Nevertheless, some researchers consider ready-to-eat cereals as a discretionary food. In the dietary supplement of the Mexican National Health and Nutrition Survey of 2012, ready-to-eat cereals have been considered a high saturated fat and/or added sugar (HSFAS) product, herein referred to as discretionary foods that should be sparingly consumed because of their high caloric content and low concentration of essential nutrients [4,51]. Another study mentioned that ready-to-eat cereals, among other processed foods, significantly contribute to the mean intake of added sugar in the Mexican diet [52]. Despite the fact that breakfast cereals are fortified, Mexican dietary guidelines do not recommend the consumption of refined cereals due the excess of possibly dangerous nutrients, such as sugar and sodium [48,53].

A longitudinal cohort study suggested that the consumption of whole-grain breakfast cereals decreased the risk of hypertension in male adults [54]. However, this study did not evaluate the consumption of processed cereals with added sugar and sodium, which might increase the risk of obesity, hypertension and other cardiovascular diseases. Furthermore, the results of Figure 1 show that the “less healthy” cereals, which are the most widely available in the Mexican market, doubled the content of added sugar and sodium compared to “healthy cereals”. The results of this study are consistent with two studies that showed that Mexican cereals are amongst the highest in sugar and sodium [26,55]. 

Our study revealed that there is a high content of added sodium in the Mexican national sample of breakfast cereals available in supermarkets (median 450 mg/100 g (SD 225.5 mg/100 g)) when using the cut-off points of the UK traffic light labelling system [56]. The United Kingdom’s sodium target for 2017 is 235 mg/100 g [57]. Australia’s target for cereals with more than 400 mg/100 g sodium is a reduction of 15% [58]. Current salt reduction strategies in Mexico are focused on reducing sodium in canned products and meats. Nevertheless, they have not considered the significant amount of sodium in breakfast cereals. Therefore, our results might urge the government to set sodium targets specifically for breakfast cereals.

In 2013, the prevalence of hypertension in Mexico reached 31.5% [59]. Therefore, the local government of Mexico City launched the campaign “Less salt, more health” to remove saltshakers from the tables of restaurants. By the end of the year, 5179 restaurants followed the campaign aiming to reduce sodium intake [60]. Additionally, the government should implement strategies to encourage the food industry to reduce sodium in processed foods. 

Our study demonstrated that products displaying claims or GDAs on the front of the package do not necessarily reflect the nutritional quality of the product. Furthermore, such labelling could mislead consumers at the time of purchase. This is consistent with literature that indicates that labelling might confuse consumers when buying a product [33,41,61]. In Mexico, a single study revealed that the GDA format was found to be confusing even for dietitian students, who were unable to correctly read the labelling information on food products [40]. An understandable FOPL system such as the traffic light or a summary indicator might be an important strategy to help consumers when choosing healthier products with less dangerous nutrients [37,38,39,40,41].

Cereals that displayed a GDA tended to have higher content of possibly dangerous nutrients. This might be explained by the fact that, at the time we conducted our research (2013–2014), the presented GDA was the result of the industry’s voluntary scheme, which used their own cut-off points. Moreover, the results of the study suggest that claims and GDAs on food packages might be used by food industries as marketing strategies to gain the attention of consumers. Some cereals (*n* = 143) did not have the GDA on the front of the package at the time of data collection, as the front of package labelling regulation had not yet been implemented by the government at that time (2013).

Since 2014, the Mexican government has introduced a mandatory FOPL strategy. The strategy consisted of placing the GDA on the front of the package. At this time, the cut-off points were established by the Ministry of Health (MoH) [62]. Despite great efforts to regulate the labelling in all food products, the cut-off points were not in line with the WHO recommendations. The cut-off points established by the MoH use 2 g of sodium as reference; moreover, they do not consider that the total intake of the population comes not only from processed foods that display labels; that non-processed foods also contribute to the sodium intake. 

Claims do not reflect the healthiness of the products. This is consistent with two studies [7,63], which showed that cereals with nutritional claims did not have better nutritional profiles than cereals without these claims. It is important to mention that all the symbolic format of claims (*n* = 14) were displayed only on “healthy cereals” category. As most of the symbolic formats are logos endorsed by the “Association of Cardiology”, this might mean that they are checking the nutritional content of their endorsed food products. 

These data might suggest the need for stronger standards in the FOPL to enable consumers to identify healthy food products, using adequate cut-off points for nutrients of interest (energy, sugar and sodium). A higher proportion of nutritional claims displayed on “less healthy” breakfast cereals suggest that stricter regulations about food labelling are needed. Mexico has the national act, NOM 086 [43], which regulates any types of claims allowed in food products. Nevertheless, compliance with this regulation is not regularly monitored. This result is consistent with other studies [64,65]. Since advertising techniques on the front of the package of food products have heavily increased, this study can set the example for other Latin-American countries to assess their food products.

### Limitations of the Study and Research Needs

This study was limited to data taken from the packaging of breakfast cereals, and does not evaluate individual consumption. Moreover, it is hard to prove the contribution of breakfast cereals to habitual nutritional intake [66,67]. It is known that individuals and populations do not consume isolated nutrients or foods as they eat combinations and food patterns determined by social, cultural and economic factors [9,22]. Another limitation of the current study was that researchers did not have the opportunity to assess the reliability of the personnel who collected the data. An interrater reliability test would have been ideal. Nevertheless, all fieldworkers were trained nutritionists, who collected the nutritional information from the packages of food products. We did not analyse the promotions and the use of characters in the package, although it is known that such factors influence the decision-making of consumers. There was possible bias when capturing the data from the photographs taken in the supermarkets. In order to decrease this possible bias, the research team established cut-off points of plausible values to eliminate outliers. Further research is needed to inform policy makers about the hazards of misleading claims, which might influence food selection and thus, could affect health. Further information is needed, as the world population is consuming higher amounts of processed foods. Research is also needed to evaluate the consumption of breakfast cereals and the nutritional quality of these cereals at the population level.

## 5. Conclusions

This study has shown that there is a lack of consistency between the labelling on the front of the pack and the nutritional quality of breakfast cereals. Mexico has taken the first steps by implementing a mandatory FOPL system for all processed foods. Nevertheless, the presence and use of a broader range of claims presents a new challenge. The current government should monitor the use of claims, set sodium targets on breakfast cereals and improve the current FOPL in addition to encouraging the food industry to follow the Mexican Regulations on labelling.

## Figures and Tables

**Table 1:** INFORMAS taxonomy about main categories and subcategories of claims [47].

Categories of Claim	Description	Subcategories and *Example*
Nutrition claims	Any representation which states, suggests or implies that a food has particular nutritional properties including but not limited to the energy value and to the content of protein, fat and carbohydrates as well as the content of vitamins and minerals.	Health-related claim *100% plant (goodness)* Nutrient claim *90 calories per serving*
Health claims	Any representation that states, suggests or implies that a relationship exists between a food or a constituent of that food and health	General health claim *Healthy eating* Nutrient and other function claim *Includes calcium, which helps build stronger teeth and bones* Reduction of disease risk claim *Lowers your blood pressure*
Other claims	Two sub claim categories have been created under the category ‘other claims’ to address claims that are not specifically related to nutrient or disease but are still heath related	Environment-related claim *Rainforest Alliance Certified* Other health-related claims *Genetically modified organism (GMOs)*

**Table 2:** Comparison of the nutritional content of breakfast cereals available in the Mexican market (*n* = 371).

	Healthy (*n* = 116) ^†^	Less Healthy (*n* = 255) ^†^	*p* Value
Nutrients	*Median (p25–p75)*	*Median (p25–p75)*	
Energy (kcal/100 g)	362.7 (343–378.3)	380 (366.6–400)	<0.001
Saturated Fat (g)	1 (0.5–1.6)	0.8 (0–1.7)	0.81
Sugar (g)	16.6 (0.3–21.4)	30.6 (24.6–37)	<0.001
Sodium (mg)	148.3 (16.6–383.3)	473.3 (393.3–600)	<0.001
UKNPM Score	0 (0–2)	9 (6–13)	<0.001
	**Claims (*n* = 282) ^§^**	**No-Claims (*n* = 89)**	***p* Value**
**Nutrients**	***Median (p25–p75)***	***Median (p25–p75)***	
Energy (kcal/100 g)	373.3 (360–397.9)	376.6 (366.6–400)	0.02
Saturated Fat (g)	1 (0–1.7)	0.7 (0–1.6)	0.50
Sugar (g)	25.4 (17.5–33.3)	30 (16.6–34.5)	0.11
Sodium (mg)	446.6 (300–533)	450 (200–566)	0.92
UKNPM Score	6 (2–11)	8 (3–11)	0.056
	GDA (*n* = 228) *	**No-GDA (*n* = 143)**	***p* Value**
**Nutrients**	***Median (p25–p75)***	***Median (p25–p75)***	
Energy (kcal/100 g)	370 (361.7–390)	388.8 (361.1–406)	<0.001
Saturated Fat (g)	1 (0.03–1.7)	1 (0–1.9)	0.21
Sugar (g)	29.6 (20–34.5)	23.2 (6.7–33.3)	0.004
Sodium (mg)	466.7 (366.7–570)	350 (83.3–466.7)	<0.001
UKNPM Score	8 (4–11.5)	5 (0–9)	<0.001

All values measured per 100 g per product; ^†^ “Healthy” and “less healthy” cereals were determined by the United Kingdom Nutrient Profiling Model; ^§^ We considered a claim according to the Codex Alimentarius definition; and * GDA: Guideline Daily Amounts.

**Table 3:** Types and format of claims displayed in breakfast cereals (*n* = 586) ^†,‡^.

		Total of Claims (*n* = 586)	Displayed Claims in “Healthy Cereals” (*n* = 274)	Displayed Claims in “Less Healthy Cereals” (*n* = 313)
Type of Claim ^§^		(%)	(%)	(%)
**Nutrition claims (*n* = 503)**		*86*	82.1	88.8
Health-related ingredient claim (*n* = 123)		*24*	22.7	25.9
	*Wholegrain (n = 54)*	44	60.8	31.9
	*Fruits/nuts/honey (n = 15)*	12	9.8	13.9
	*Grains/seeds (n = 26)*	21	19.6	22.2
	*Cereals (n = 26)*	21	5.9	31.9
	*Probiotics (n = 2)*	2	3.9	0.0
Nutrient content claim (*n* = 355)		71	70.7	70.5
	*Fiber (n = 66)*	19	27.0	11.7
	*Energy (n = 5)*	1	0.0	2.6
	*Antioxidants/vitamins/minerals (n = 195)*	55	39.0	67.9
	*Fats (n = 34)*	10	8.2	10.7
	*Sugar (n = 11)*	3	6.3	0.5
	*Calcium (n = 3)*	1	1.9	0.0
	*Protein (n = 24)*	7	13.8	1.0
	*Salt (n = 2)*	1	1.3	0.0
	*Cholesterol (n = 12)*	3	1.3	5.1
	*Omega 3 (n = 3)*	1	1.3	0.5
Nutrient comparative claim (*n* = 25)		5	6.7	3.6
	*More calcium (n = 1)*	4	0.0	10.0
	*Reduced sugar (n = 7)*	28	13.3	50.0
	*More fiber (n = 3)*	12	0.0	30.0
	*Splenda sweetened (n = 14)*	56	86.7	10.0
**Health claims (*n* = 23)**		*4*	4.4	3.5
General health claim (*n* = 11)		*48*	33.3	63.6
	*General/healthy (n = 11)*	100	100.0	100.0
Nutrient and other function claim (*n* = 7)		*30*	25.0	36.4
	*Calcium or Vitamin D for bone (n = 6)*	*86*	100.0	75.0
	*Nutrient+digestion (n = 1)*	14	0.0	25.0
Reduction of disease risk claim (*n* = 5)		*22*	41.7	0.0
	*Heart-related (n = 2)*	40	40.0	0.0
	*Cholesterol absorption (n = 3)*	60	60.0	0.0
**Other claims (*n* = 61)**		10.4	13.5	7.7
Other health related claim (*n* = 55)		90.2	83.8	100.0
Environment claim (*n* = 6)		9.8	16.2	0.0
**Format of claim**				
Numerical (*n* = 155)		26.4	26.6	26.2
Written text (*n* = 418)		71.2	68.2	73.8
Symbolic (*n* = 14)		2.4	5.1	0.0
GDA *			22.8	77.2

^†^ Total (*n* = 371) cereals. ^‡^ each product could carry more than one claim. ^§^ We stratified the claims according to the INFORMAS taxonomy. * GDA: Guideline Daily Amounts.
